# Applications of CRISPR-Cas9 for advancing precision medicine in oncology: from target discovery to disease modeling

**DOI:** 10.3389/fgene.2023.1273994

**Published:** 2023-10-16

**Authors:** Mirunalini Ravichandran, Danilo Maddalo

**Affiliations:** Department of Translational Oncology, Genentech, Inc., South San Francisco, CA, United States

**Keywords:** CRISPR/Cas9, CRISPRa/i, precision oncology, epigenome editing, base editing, prime editing, cancer modeling, CRISPR screening

## Abstract

The clustered regularly interspaced short palindromic repeats (CRISPR) and CRISPR-associated protein 9 (Cas9) (CRISPR/Cas9) system is a powerful tool that enables precise and efficient gene manipulation. In a relatively short time, CRISPR has risen to become the preferred gene-editing system due to its high efficiency, simplicity, and programmability at low costs. Furthermore, in the recent years, the CRISPR toolkit has been rapidly expanding, and the emerging advancements have shown tremendous potential in uncovering molecular mechanisms and new therapeutic strategies for human diseases. In this review, we provide our perspectives on the recent advancements in CRISPR technology and its impact on precision medicine, ranging from target identification, disease modeling, and diagnostics. We also discuss the impact of novel approaches such as epigenome, base, and prime editing on preclinical cancer drug discovery.

## Introduction

The clustered regularly interspaced short palindromic repeats (CRISPR) and CRISPR-associated (Cas) protein (CRISPR/Cas) system is an inheritable adaptive immune system found in both archaeal and bacterial organisms. It is broadly classified into two major classes encompassing six types and several subtypes (Zhu, 2022; Mishra et al., 2023). Within this system, class I utilizes multiple effector proteins, while the class II system employs a single effector protein for target interference. One of the most well-characterized and commonly used systems within the CRISPR/Cas family is the type II, class II CRISPR system that relies on a single Cas9 protein (Zhu, 2022; Mishra et al., 2023). Mechanistically, the induction of double-stranded DNA break (DSB) is achieved by the binding of Cas9 endonuclease to the target DNA sequence guided by the single-guide RNA (sgRNA), a fusion of trans-activating RNA (tracrRNA) and CRISPR-targeting RNA (crRNA). For the DNA cleavage to be precise and highly specific, the target DNA sequence should include an “NGG” protospacer adjacent motif (PAM) located upstream of the 3′-end of the target sequence. DSB is resolved mainly by two cellular DNA repair pathways, namely, non-homologous end joining (NHEJ) and homology-directed repair (HDR). NHEJ is an error-prone process that introduces insertions or deletions resulting in gene disruption by shifting the reading frame. In contrast, HDR can introduce precise DNA changes using the DNA template containing homologous arms (Anzalone et al., 2020; Zhu, 2022; Mishra et al., 2023). Due to the simplicity of the system, flexibility in PAM requirement that allows for targeting a wide range of DNA sequences, and high precision in genome editing, CRISPR/Cas9 has been widely adopted as the “go-to” tool for gene editing.

**FIGURE 1:**
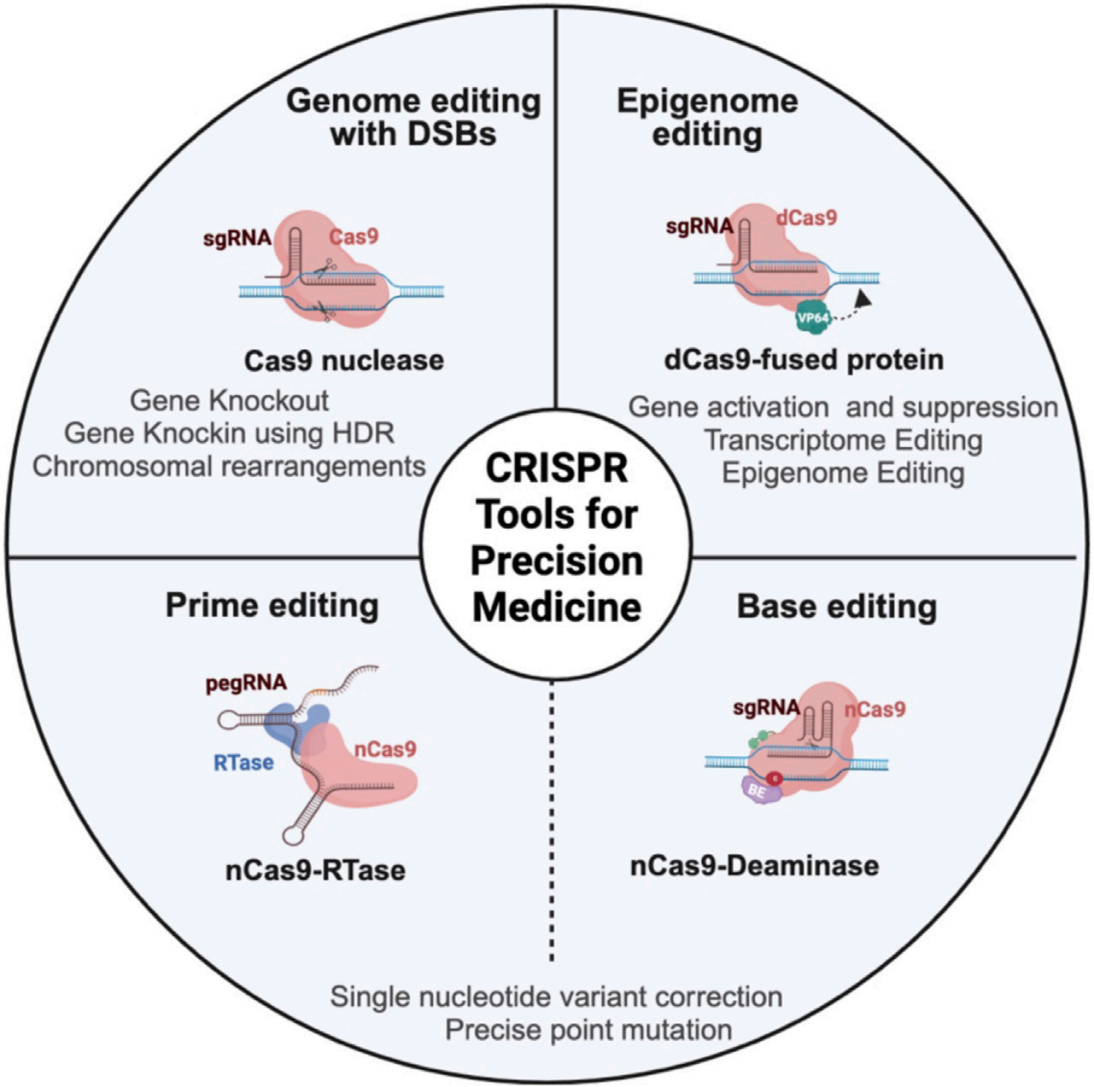
Schema representing different CRISPR-based genome and epigenome engineering tools in cancer research. CRISPR/Cas9 nuclease achieves gene editing by a programmable single-guide RNA (sgRNA), which is a fusion of trans-activating RNA (tracrRNA) and CRISPR-targeting RNA (crRNA), to guide the Cas9 protein to the target DNA. Once the Cas9 protein recognizes the DNA, it will induce double-stranded break (DSB), which is resolved mainly by error-prone non-homologous end joining (NHEJ) pathway or homology-directed repair (HDR), which is a more precise repair mechanism to introduce specific changes to the DNA. The epigenome editor contains the dead Cas9 (dCas9) protein fused to epigenetic effector proteins. Similarly, the transcriptome editor has the dCas9 protein fused to transcriptional activators or repressors. Both epigenome and transcriptome editors modulate the chromatin and transcriptome without altering the underlying DNA sequence. The base editor utilizes a mutant Cas9 nickase (nCas9) fused to deaminase (cytosine or adenine deaminase). nCas9 introduces a nick in the non-edited strand that induces cellular machinery to modify the non-edited strand based on the edited template. The base editor introduces a single-base mutation at the target locus without creating DSB. The prime editor contains Cas9 nickase fused to reverse transcriptase (RTase) and prime-editing guide RNA (pegRNA). PegRNA is an engineered RNA that contains the sequence that targets the prime editor to the target DNA and the sequence that serves as a template for desired DNA sequence change. Prime editing can introduce indels and point mutations without introducing double-stranded break.

**FIGURE 2:**
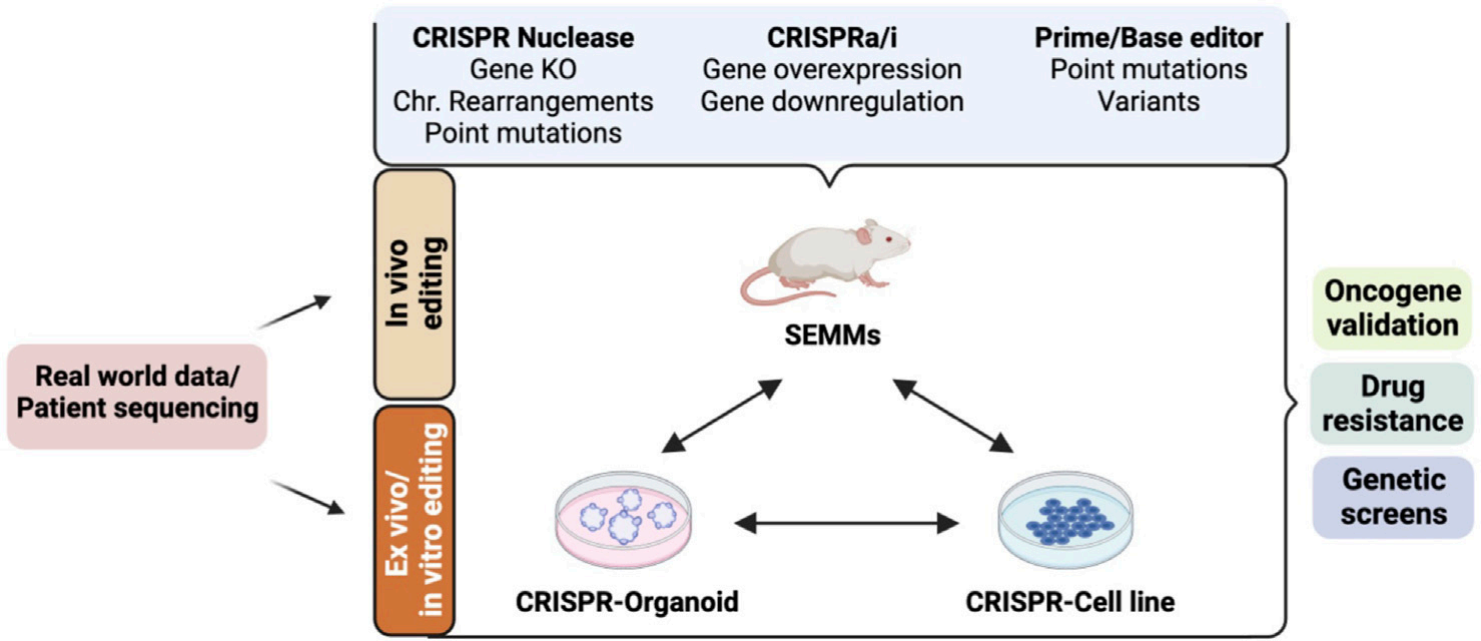
Cellular and animal models established using the CRISPR/Cas9-based gene-editing system and their applications in preclinical cancer drug discovery. Real-world patient datasets can be used to generate *in vitro* (cellular and organoid) or *in vivo* models such as somatically engineered mouse models (SEMMs) using CRISPR/Cas9 variants. CRISPR nuclease-mediated gene editing can be used to generate models carrying gene knockout, gene knock-in, chromosomal (Chr.) rearrangements, or point mutations using the homology-directed repair mechanism. CRISPR activation (CRISPRa: fusion of dead Cas9 protein and transcriptional activators) and/or CRISPR interference (CRISPRi: fusion of dead Cas9 protein and transcriptional repressors) can be used to generate models with gene(s) of interest activated and/or repressed. Base and prime editors can be used to generate models carrying a precise gene mutation. These models can be used for various applications in preclinical cancer research, such as drug sensitivity/resistance, oncogene identification and validation, mechanism of action of a drug, genetic screen to identify cancer-specific dependencies, and synthetic gene lethality.

Since its adaptation as a programmable gene editing tool in mammalian cells, the CRISPR/Cas9 system has transformed the landscape of genome engineering (Jinek et al., 2012; Cox et al., 2015). Its cost-effectiveness, facile design, and high efficiency have enabled it a powerful and preferred gene editing tool with enormous potential in both basic research and drug discovery platform. This is particularly true in cancer research, as CRISPR has had a huge impact on our understanding of cancer biology and many phases of cancer drug development (Yin et al., 2019; Long et al., 2021; Katti et al., 2022a; Lattanzi and Maddalo, 2022). For instance, its fast and efficient genome alterations have uncovered novel cancer-specific gene mutations and enabled rapid screening to identify and validate potential drug targets, precise disease modeling, and development of cancer therapeutics (Katti et al., 2022a). Moreover, recent CRISPR-based innovations such as base editing and prime editing have expanded the scope and capabilities even further by enabling precise genome manipulation at single-base resolution (Komor et al., 2016; Gaudelli et al., 2017; Rees and Liu, 2018; Anzalone et al., 2019; Chen and Liu, 2023). In addition to gene editing, CRISPR can be employed to modulate gene expression and interrogate non-coding elements through CRISPR interference (CRISPRi) and CRISPR activation (CRISPRa)-based epigenome and transcriptome editing, thus providing an added flexibility in the way we reprogram our genome (Goell and Hilton, 2021; Gilbert, 2022). These innovations hold enormous potential in creating effective and personalized treatment strategies. In this review, we discuss the latest developments in CRISPR technology (Figure 1), emphasizing its application in advancing precision medicine, with a main focus on preclinical cancer research. We also describe how emerging technologies such as epigenome, prime, and base editing open up new avenues for precision oncology and continue to accelerate basic cancer research, preclinical cancer drug discovery, diagnosis, and treatment (Figure 2; Table 1). Furthermore, we briefly discuss the prevailing challenges and limitations in the practical application of this technology.

**TABLE 1:** Table summarizing different CRISPR/Cas9 variants and their application in precision disease modeling.

Cas9 variants	Components	Effector domain(s)	Gene edits	Applications
CRISPR/Cas9 nuclease	Cas9 endonuclease and sgRNA	None	Gene knockout with indels	High-throughput functional genomics screen in *in vitro* (cellular and organoid models), *ex vivo*, and *in vivo* systems
			Gene knock-in using the exogenous HDR template	*In vitro* and *in vivo* disease modeling (knock-in and knockout models)
			Introduce DSB	Cancer diagnostics
Epigenome editor	dCas9 fused to epigenome editors and sgRNA	Epigenome editors such as TET enzymes, DNMT3A, 3L, and MECP2	Chromatin reorganization	Complex genetic modeling *in vitro* as well as *in vivo* albeit less efficiency
			Histone modifications	
			DNA methylation and demethylation	
			Non-coding elements	
			Does not introduce DSB	
Transcriptome editor	Dead Cas9 fused to gene activators (CRISPRa) or gene suppressors (CRISPRi) and sgRNA	VP64, VP16, and KRAB domains	Transcriptional activation and repression	High-throughput functional genomics screen in *in vitro*, *ex vivo*, and *in vivo* systems
			Does not introduce DSB	
Base editor	Cas9 nickase fused to adenine or cytosine base editor and sgRNA	Adenine deaminase (example: Tad-8e); cytidine deaminase (example: AID/APOBEC)	Single-nucleotide editing	Modeling SNVs *in vitro* and *in vivo*
			ABE: A to G	
			CBE: C to T	High-throughput SNV screening
			Introduces DNA nick	
Prime editor	Dead Cas9 fused to reverse transcriptase to pegRNA	Reverse transcriptase	All 12 base substitutions	Modeling SNVs
			Short insertion and deletion without donor plasmid and DSB	Modeling gene knock-in or mutant with single/multiple mutations
			Requires modified sgRNA	

sgRNA, single-guide RNA; pegRNA, prime-editing RNA; dCas9, dead Cas9; DSB, double-stranded break; SNVs, single-nucleotide variants; ABE, adenine base editor; CBE, cytosine base editor; AID/APOBEC, activation-induced cytidine deaminase/apolipoprotein B mRNA-editing enzyme catalytic polypeptide-like; DNMTs, DNA methyltransferases; KRAB, Krüppel-associated box domain; MECP2, methyl-CpG-binding protein 2; TET, ten-eleven translocation.

## CRISPR as a tool in target discovery in cancer

### Screening using CRISPR nuclease

Identifying cancer-specific vulnerabilities (genetic and pharmacological dependencies) that can be exploited clinically is a major goal in cancer research. While this may sound simple, it is quite challenging given the genetic complexity of the disease. However, due to its ease of design, multiplexing ability, and high efficiency, CRISPR/Cas9-based pooled genetic screening has rapidly emerged as a powerful tool for interrogating large-scale functional genomics and identifying cancer dependencies (Katti et al., 2022a). Several high-throughput CRISPR-based screenings (both genome-wide and focused) have successfully uncovered genetic vulnerabilities as well as genes that mediate drug response (sensitivity or resistance) in a wide variety of cancer cell lines (Hart et al., 2015; Krall et al., 2017; Przybyla and Gilbert, 2022). For instance, a CRISPR/Cas9 deletion screen performed in the presence of inhibitors targeting the RTK/MAPK pathway in lung cancer cells identified the loss of KEAP1 as a key factor in conferring resistance and promoting cell survival (Krall et al., 2017). Another example is the viability-based CRISPR knockout screen performed in around 800 cancer cell lines known as the cancer dependency map (DepMap). DepMap provides a valuable source of information in identifying gene essentiality and gene dependencies in specific cancer types (Meyers et al., 2017; Behan et al., 2019). This information can be leveraged to prioritize cancer drug targets, develop new cancer-specific targets, validate previously identified drug targets, identify biomarkers associated with drug sensitivity/resistance, and enable rational drug combinations. In addition to gene essentialities, DepMap enables researchers to uncover novel metabolic pathways, protein complexes, and gene pairs that exhibit synthetic lethality in cancer (Przybyla and Gilbert, 2022). Moreover, screening approaches that exploit in-frame mutations created by CRISPR-induced non-homologous end joining (NHEJ) in essential genes can be used to identify mechanisms of action of anticancer agents and identify novel protein variants that confer resistance to drug treatments (Donovan et al., 2017; Neggers et al., 2018). For instance, a CRISPR/Cas9-mediated mutagenesis screen identified variants of the MAPK pathway genes, *MEK1* and *BRAF1*, that are resistant to the inhibitors selumetinib and vemurafenib, respectively, in melanoma cell lines (Donovan et al., 2017).

Advanced screening strategies that employ CRISPR to systematically map high-resolution lethal gene interactions in several cancers have been reported (Anderson et al., 2017; Shen et al., 2017; Han et al., 2020; Thompson et al., 2021). For example, in cancers with KRAS-mutant—a key oncogenic driver found in colon, ovary, lung, and pancreas cancers—screening performed using CRISPR nuclease identified synthetic lethal dependencies whose loss cooperates with and sensitizes cancer cells to the inhibition of KRAS pathway effectors (Anderson et al., 2017). Moreover, integrating CRISPR functional genomics data with genetic mutation information can be valuable in identifying patient- or mutant-specific synergistic drug targets and prioritizing cancer treatment. For instance, through the DepMap database search, Werner syndrome ATP-dependent helicase (WRN DNA helicase) was identified as a synthetic lethal gene in different cancer cells that harbor mutations in DNA mismatch repair (Behan et al., 2019; Chan et al., 2019). Furthermore, pooled CRISPR screens can be combined with single-cell RNA-Seq (Perturb-Seq) to map high-resolution transcription data on cellular response to specific CRISPR manipulations (Dixit et al., 2016). This is a powerful method to understand how individual gene perturbations affect cellular pathways and disease progression. These studies highlight how modularity of CRISPR/Cas9 screens can be combined with genomic and transcriptomic data to gain insights into functional genomics that contribute to more effective targeted therapies in cancer.

With recent advancements in 3D culture, it is now possible to identify cancer-specific genetic vulnerabilities in organoids through large-scale screens. Recently, a CRISPR screen focusing on tumor suppressor genes (TSGs) in a pre-malignant colon organoid treated with TGF-β inhibitor identified genes that inhibit tumor growth (Michels et al., 2020). In addition to *in vitro* screening, it is feasible to perform *in vivo* screening, either through transplantation of *ex vivo* engineered cell lines/organoids into mice (Michels et al., 2020) or through direct delivery of the CRISPR components into somatic cells (Chow et al., 2017; Lima and Maddalo, 2021). For instance, Chen et al. (2015) transplanted an *ex vivo* transduced genomic-wide library into an immunocompromised mouse to identify potential regulators of metastasis in non-small-cell lung carcinoma (NSCLC). In another study, the authors delivered adeno-associated virus (AAV) carrying sgRNA, targeting most commonly mutated cancer genes directly into the mouse brain to identify drivers and co-drivers in glioblastoma tumors (Chow et al., 2017). These studies suggest that CRISPR screens continue to improve our understanding of how genes and pathways contribute to the development of cancer. While *in vitro* screens provide novel insights into cancer-specific genetic, metabolic, and growth dependencies, *in vivo* screening accounts for the complexity of the tumor microenvironment, thus having the potential to identify more effective therapeutic targets (Chow and Chen, 2018; Kuhn et al., 2021). Moreover, they are useful in situations where tumor cells could not be cultured easily, for instance, in patient-derived xenograft (PDX) cell lines (Hulton et al., 2019).

### Screening using CRISPRi/CRISPRa

Despite being a valuable tool, CRISPR knockout screens have several limitations. First, they only allow for identifying genes that are essential for cell survival but not necessarily those with a subtle phenotype or response to treatment. Another limitation is the potential for high false-positive hits, especially when perturbing highly amplified regions (Munoz et al., 2016). Furthermore, they may not fully recapitulate the complex genetic and epigenetic phenotypes that occur in a tumor. These limitations can be overcome by CRISPRi and CRISPRa platforms which allow for precise and subtle modulation of gene expression (Gilbert et al., 2013; Heidersbach et al., 2022; Przybyla and Gilbert, 2022).

CRISPRi achieves robust gene inactivation by utilizing nuclease-dead Cas9 (dCas9) fused to transcriptional repressors such as KRAB, while CRISPRa involves the fusion of dCas9 to transcriptional activators such as VP64 to activate gene expression (Gilbert et al., 2013; Gilbert et al., 2014). Several other versions of dCas9 fused to diverse effector domains, such as methyltransferases and histone writers, enable reversible and tunable genome and epigenome modulation at any desired locus (Nakamura et al., 2021).

Since the introduction of CRISPRi and CRISPRa, several groups have uncovered novel cancer-specific vulnerabilities in various cancer cell lines by utilizing these technologies in functional genomic screens (Kampmann, 2018; Gilbert, 2022). For instance, through CRISPRi screening, Lou et al. identified collateral dependencies (reliance on an alternate pathway upon inhibition of oncogene) to the inhibition of the oncogene, KRAS G12C, in lung and pancreatic cancer cells. They identified that the combined inhibition of KRAS G12C together with SHP2, CDK4/6, or EGFR further sensitizes the cells to KRAS G12Ci (Gilbert et al., 2013; Lou et al., 2019). Moreover, they have the potential to uncover novel genes that were not previously identified through CRISPR knockout approaches. This is particularly true for those genes where partial knockdown of genes affects cell proliferation or viability, as CRISPRi partially suppresses their expression rather than ablating them (Sage et al., 2017). Additionally, CRISPRi screens are more suited for identifying drug targets than CRISPR knockout screens because drugs usually decrease target’s function rather than completely eliminating it (Sage et al., 2017). Another advantage of CRISPRi screens over CRISPR knockout screens is that they can be multiplexed without the concern of inducing DSBs (Gilbert et al., 2013).

Similarly, CRISPRa-based screening holds a great potential in elucidating resistance mechanisms which are believed to often result from gain-of-function phenotypes. For instance, CRISPRa screening in a V600E melanoma cell line treated with BRAF inhibitors revealed previously-known as well as novel gene components that confer resistance to the inhibitor (Konermann et al., 2015). Moreover, with CRISPRa, it is feasible to probe alternate transcription start sites in the context of a screen (Kampmann, 2018). The reversible and tunable nature of CRISPRi and CRISPRa screens has established them as the tool of choice for interrogating the function of non-coding elements such as lncRNAs and studying the effect of the promoter–enhancer interaction on gene expression (Morgan et al., 2017; Gasperini et al., 2019; Liu et al., 2020). A significant advantage of using dCas9-based gene editing is the ability to perform complex gene manipulations. For instance, by using orthogonal Cas systems for CRISPRi or CRISPRa, multiple genes can be activated and silenced simultaneously within the same cell, thus facilitating the study of directional interactions (Boettcher et al., 2018).

However, there are several limitations associated with CRISPRi and CRISPRa. The genome and epigenome changes mediated by the dCas9 platform are reversible and may not be durable. Several ongoing efforts are being made to address this issue by recruiting multiple effectors such as a combination of epigenome and transcriptional regulators. One example of this is the use of dCas9 fused to KRAB-DNMT3A-DNMT3L to enforce robust and heritable gene modulation (Nuñez et al., 2021). Additionally, for the toolkit to be effective, the effector combination must be optimized for the individual cell and tissue type, and chromatin accessibility should be considered. Furthermore, modulating gene expression using CRISPRa is not effective when there are loss-of-function mutations in the coding region (Gilbert, 2022).

### Screening using base editors

In addition to CRISPR nuclease and CRISPRi/a screens, it is now possible to screen for single-nucleotide variants (SNVs) in a high-throughput manner using base editing which consist of a mutant Cas9 nickase (nCas9) fused to deaminase (cytosine or adenine deaminase) (Gaudelli et al., 2017). By precisely installing a single-base mutation at the target locus, base editor screening allows for the identification and characterization of disease-associated variants in cancer, thus pinpointing the phenotypic effects resulting from that specific mutation. Additionally, it is effective in identifying gene variants that mediate sensitivity or resistance to drugs (Cuella-Martin et al., 2021; Hanna et al., 2021; Kim et al., 2022; Coelho et al., 2023). For instance, Coelho et al. (2023) used the base-editing mutagenesis screen targeting the JAK-STAT pathway to identify genetic variants that mediate sensitivity and resistance to interferon gamma (IFNγ) response in colorectal cancer (CRC). Base editor screenings are useful in the drug discovery process, especially to identify variants that influence the drug–protein interaction. For instance, the sgRNA tiling approach using base editors could be used to identify potential gene mutations that alter the drug–protein interaction and confer resistance. Therefore, it is possible to use this platform to predict beforehand whether a particular variant can impart resistance to drug treatment (Hanna et al., 2021). Moreover, base editor-mediated introduction of variants in the catalytic domain of a potential drug target could mimic small-molecule inhibition and serves as a better alternative than knockdown or knockout approaches. Overall, base editor screening has opened up new promising possibilities to identify and study functional consequences of genetic variants, making it a valuable tool in precision medicine.

The versatility of CRISPR technologies has provided a range of screening options. However, each screening platform has its own capabilities and limitations. Therefore, it is crucial to evaluate the specific research objective when selecting a screening method to ensure success. Moreover, the identified targets should be thoroughly validated using orthogonal approaches to increase confidence in them.

## CRISPR as a tool in precision cancer modeling

Advancements in sequencing technology and genome-wide association studies (GWAS) have yielded an extensive list of genetic mutations for various types of cancer. These patient-driven datasets are instrumental in precision medicine, aiding in the identification of biomarkers and devising effective personalized treatment strategies for cancer patients. However, identifying the mutations that are causally linked to tumorigenesis remains a significant challenge. Therefore, it is important to characterize the function of these mutations in relevant model systems to assign causality and to identify actionable targets for therapeutics. CRISPR-mediated gene editing has had a profound impact on cancer modeling. CRISPR knockout and knock-in strategies have been quickly adapted in the generation of cellular and animal models. Another exciting new development in precision disease modeling is the introduction of base and prime editors that can directly introduce disease-specific gene mutation without the need for the DNA template (Komor et al., 2016; Gaudelli et al., 2017; Rees and Liu, 2018; Chen and Liu, 2023). These models serve as valuable tools in identifying and validating patient-specific cancer drivers and resistance mechanisms (Smurnyy et al., 2014; Yin et al., 2019; Katti et al., 2022b).

### *In vitro* cancer modeling using CRISPR/Cas9

With CRISPR, establishing cellular cancer models becomes simple and straightforward. These cellular models are fast to generate, cost-effective, multiplexable, and scalable, making them suitable for high-throughput screening. They have been widely used for mechanistic analysis, gene interaction mapping, and target validation in different cancer types (Jalaleddine et al., 2019; Christodoulou et al., 2021; Jefremow et al., 2021; Akram et al., 2022; Przybyla and Gilbert, 2022). For instance, Zhang et al. (2019) used CRISPR/Cas9 in gastric cancer cell lines to study the role of the transcription factor, prostate-derived Ets factor (PDEF), in promoting tumorigenesis. In another study, Jalaleddine et al. (2019) showed that deletion of pannexin-1 (PANX1) using CRISPR/Cas9 in MDA-MB-231 cells promotes breast cancer metastasis through positive regulation of epithelial-to-mesenchymal transition (EMT) genes. Wohlhieter et al. (2020) employed a CRISPR knockout strategy to establish a cellular model for lung adenocarcinoma (LUAD) to identify cancer-specific metabolic dependency. They discovered an increased reliance on the metabolic gene, *stearoyl-CoA desaturase 1* (*SCD1*), in cells after deletion of the tumor suppressor genes, *serine/threonine kinase 11* (*STK11*) and *Kelch-like ECH-associated protein 1* (*KEAP1*), that are co-mutated in ∼10% of LUAD patients and are associated with an aggressive phenotype. Importantly, the increased dependence on *SCD1* enables cell survival by imparting protection against ferroptosis, suggesting *SCD1* as a potential drug target. CRISPR/Cas9 can be used to define selective resistance mechanisms in response to drug treatments. For instance, Hussmann et al. (2017) employed CRISPR-mediated gene deletion to investigate the mechanism of acquired resistance to the EGFR inhibitor, erlotinib, in HCC827 lung cancer cells. The findings revealed that the deletion of the insulin-like growth factor 1 receptor (IGF1R) promotes MET amplification and reduces the mesenchymal level, leading to acquired resistance to erlotinib in these cells. These studies and numerous other studies (Ramkumar and Kampmann, 2018; Jalaleddine et al., 2019; Christodoulou et al., 2021; Akram et al., 2022; Przybyla and Gilbert, 2022) underscore the invaluable role of 2D cellular models in advancing our understanding of cancer biology and facilitating drug development.

While cell lines serve as a robust model, prolonged passaging of cell lines may not faithfully reflect the biology and pathophysiology of the original tumor. Moreover, they fail to fully capture the complexity of the disease. For instance, it is difficult to model the interaction of cancer cells with their microenvironment or immune cells. These limitations can be partially overcome by the utilization of a 3D organoid culture system that is robust and recapitulates many important molecular and phenotypic characteristics of tumors. Several studies have utilized CRISPR to generate organoid models carrying defined cancer mutations (patient-specific) to characterize drivers and to test therapies (Roper et al., 2017; Lo et al., 2021). Recently, CRISPR-based knockout in a gastric organoid model identified the context-dependent role of AT-rich interactive domain-containing protein 1A (ARID1A) in early tumorigenesis and uncovered ARID1A knockout-dependent therapeutic vulnerability (Lo et al., 2021). Other groups have exploited CRISPR’s multiplexing ability in generating complex organoid models (lung, colon, and breast) that carry several mutations to identify oncogenic drivers and to test drug response (Matano et al., 2015; Drost et al., 2017; Dekkers et al., 2019). For instance, by generating intestinal organoids that carry four commonly mutated colon cancer genes, Drost et al. (2017) identified that losing APC and P53 is sufficient to drive tumorigenesis *in vivo*. Given the advantages offered by 3D culture (its ability to mimic tumor heterogeneity, aspects of *in vivo* microenvironment, and providing physiologically relevant drug response), they could serve as a better and relatively cheaper alternative in the drug discovery process to screen and validate potential cancer drivers and resistance genes. Interestingly, a recent work reported that cancer dependencies identified from a 3D spheroid model closely resemble *in vivo* tumor, thus emphasizing the potential of 3D culture as a valuable tool in cancer research (Han et al., 2020).

### *In vivo* cancer modeling using CRISPR/Cas9

CRISPR has made a significant impact on the generation of *in vivo* models. Given the complex genetic landscape of tumors, the mouse serves as an effective model in determining how context-specific genetic mutations influence tumorigenesis and reveal tumor-specific genetic vulnerabilities. Additionally, mouse models can be used to test the efficacy and safety of a potential drug. The advent of CRISPR has enabled faster generation of precise and complex animal models, thus reducing the timeline of cancer drug discovery. Mouse models can be generated by the introduction of the CRISPR components either in blastocysts (Zafra et al., 2020) or directly in the somatic tissue of interest (Lima and Maddalo, 2021). In particular, the latter method provides a swift approach to generating somatically engineered mouse models (SEMMs) (Lima and Maddalo, 2021). Several studies have generated mouse knockout models with complex genetic phenotypes by delivering CRISPR components into lungs (Sánchez-Rivera et al., 2014; 2022), liver (Xue et al., 2014), or pancreas (Chiou et al., 2015; Ideno et al., 2019). In addition to knockout models, HDR-mediated CRISPR knock-in was used to model single-nucleotide variants (SNVs), albeit with less efficiency (Winters et al., 2017). For instance, several groups have utilized the CRISPR knock-in strategy using HDR to generate mutant KRAS-driven mouse models to study its contribution to tumorigenesis (Winters et al., 2017; Zafra et al., 2020). One such study identified a distinct tissue and tumor-specific role for specific KRAS variants in tumor initiation and progression, emphasizing the need to model precise gene mutations observed in human cancers (Winters et al., 2017). In another study, the authors engineered different KRAS variants in colon, lung, and pancreas of the mice using HDR-mediated CRISPR editing. Characterization of distinct KRAS mutation uncovered significant differences in tumor initiation and progression. It also revealed KRAS mutant-specific therapeutic vulnerabilities, again underscoring the need to study individual cancer driver mutants to understand distinct phenotypes and to develop effective mutant-based treatment strategies (Zafra et al., 2020). Moreover, DSBs induced by Cas9 can be utilized to model complex chromosomal rearrangements that mimic oncogenic gene fusions found in patients. This is achieved by targeting two chromosomes of interest using two gRNAs. Using viral-mediated delivery of CRISPR/Cas9 components into mouse lungs, Maddalo et al. (2014) successfully achieved the fusion of the genes, *echinoderm microtubule*-*associated protein*-*like 4* (*EML4*) and *anaplastic lymphoma kinase* (*ALK*). This method generated EML4–ALK oncogenic gene fusion, an oncogene detected in a subset of NSCLC, that can be used to study EML4–ALK fusion-driven lung tumor.

CRISPR offers the flexibility of target multiplexing and can be used to generate complex mouse models (Weber et al., 2015; Maresch et al., 2016). For instance, multiple gRNAs were delivered into the mouse pancreas of the KRAS G12D mouse model to study the synergistic gene interaction that drives tumorigenesis (Maresch et al., 2016). This is extremely useful in understanding how a set of genes coordinates with each other to control a complex phenotype. However, there is an imminent risk of toxicity induced by DSBs when targeting multiple genes using CRISPR nuclease. These issues can be addressed by the use of dCas9-based transcriptome and epigenome editing (Goell and Hilton, 2021; Rahman and Tollefsbol, 2021). For instance, Zhou et al. (2018) used epigenome editing to simultaneously activate 10 genes within the mouse brain with high specificity and minimal off-target effects. Likewise, many other studies applied epigenome and transcriptome editing in mice to characterize complex genetic and epigenetic determinants of cancer (Goell and Hilton, 2021; Rahman and Tollefsbol, 2021). For example, Li et al. (2020a) used multiple epigenetic effectors to stably perturb enhancer activity *in vivo*. They showed that allele-specific perturbation of oncogenic super-enhancer T-cell acute lymphoblastic leukemia (T-ALL) induces mRNA and protein expression and promotes tumor progression (Li et al., 2020a). Despite its great potential in modeling and treating diseases, the efficiency of epigenome modulating CRISPR technology in generating stable and heritable changes is relatively low *in vivo* and needs to be improved.

In addition to mouse, zebrafish has emerged as a reliable and robust preclinical cancer model. This is mainly due to its evolutionary conservation in the genetic sequence with human, its short life cycle, high reproductive rate (i.e., ability to produce hundreds of progenies in single mating), transparent embryo, embryonic development outside the uterus, and cost-effectiveness in maintaining and performing experiments in a large cohort of animals (Hason and Bartůněk, 2019; Patton et al., 2021). Recent studies have revealed that zebrafish shares 70% sequence homology with human, indicating structural and functional similarities between human and zebrafish genes as well as the molecular mechanisms and pathways. Furthermore, ∼82% of human disease-related genes have an ortholog in zebrafish, making it a valuable and reliable alternative for precision disease modeling (Chen et al., 2021; Patton et al., 2021). Zebrafish can develop spontaneous tumors that resemble human cancer in terms of genetics, morphology, and signaling pathways (Rubbini et al., 2020). This makes it an ideal model for exploring disease mechanism, tumor drivers, metastasis, and response to therapy (Chen et al., 2021; Patton et al., 2021).

Advances in CRISPR gene editing technologies combined with high-level imaging and ease of genetic manipulation have opened up new possibilities in precision cancer modeling in zebrafish (Raby et al., 2020; Patton et al., 2021). For instance, by using CRISPR-mediated gene editing in zebrafish, Ablain et al. (2018) defined the *sprouty-related EVH1 domain*-*containing* 1 (*SPRED1*) gene, a negative regulator of the RAS-MAPK pathway, as a potent tumor suppressor in mucosal melanoma. They found that its loss resulted in early onset and rapid progression of KIT-driven mucosal melanoma (Ablain et al., 2018). CRISPR-based gene editing also enabled rapid generation of complex zebrafish genetic models such as introducing several oncogenic mutations and tumor suppressor genes in different organs in a tissue-specific manner (Ablain et al., 2015; 2018). CRISPR has been used to rapidly generate all major genotypes found in melanoma in a melanocyte-specific manner, and these mutant fish formed tumors within weeks or months, which is faster than the previous models that used the similar approach (Callahan et al., 2018). Moreover, thanks to CRISPR technology, performing complex and selective gene edits in the tumor microenvironment in zebrafish becomes more accessible (Liu et al., 2019). Recently, base editing technology has been applied to precisely model single-base mutation in zebrafish, which opens up exciting possibilities in conducting targeted genetic studies and disease modeling in this versatile model organism (Rosello et al., 2021; Rosello et al., 2022; Rosello et al., 2023). Zebrafish has also become an attractive model for xenograft transplantation (Patton et al., 2021). Transplanting cancer cells carrying patient-specific mutation or PDX in zebrafish facilitates the testing of therapy response (Fior et al., 2017; Almeida et al., 2020). Numerous studies have explored the zebrafish xenografts in various cancer types (Patton et al., 2021). Due to its transparent body, it is easy to monitor the growth and dynamics of cancer *in vivo*, especially in studying spontaneous metastasis, which is a complex and multistep process (Weiss et al., 2022).

Another key advantage of using zebrafish is that they respond to drug treatments at physiologically relevant doses, which makes it a powerful model to study phenotypic drug screening (Hason and Bartůněk, 2019). These traits combined with cost-effectiveness make zebrafish an ideal platform for assessing drug response in the context of personalized treatments. Despite these advantages, there are several limitations associated with the zebrafish model, such as body temperature difference, limited organ complexity, and variability in tumor incidence and growth (Hason and Bartůněk, 2019). Each model organism has its own strength and weakness. Therefore, it would be beneficial to combine different model systems to gain a deeper understanding of cancer biology and to develop targeted therapies.

### Modeling using base and prime editors

With the advent of base editing, modeling SNVs has become much easier. Its high efficiency in introducing precise point mutation, without introducing DSB or requiring a donor template, makes it an ideal tool for cancer drug discovery, as most of the cancer variants are point mutations. Within a relatively short time since its development, base editing has been successfully implemented in the generation of cellular, organoid, and animal models (Komor et al., 2017; Musunuru et al., 2021; Rothgangl et al., 2021; Li et al., 2022a; Katti et al., 2022b). For instance, Li et al. (2020b) used an adenine base editor to successfully correct oncogenic point mutations on the TERT promoter in glioblastoma cells, leading to tumor growth inhibition. In another study, Sayed et al. (2022) used base editing technology to correct oncogenic KRAS and TP53 mutations in cancer cell lines and patient-derived organoids. They showed that targeting KRAS oncogenic mutation decreases tumor growth in various cell lines and organoids. These studies underscore the potential of base editors in advancing personalized treatment in the context of precision oncology. In addition to *in vitro* models, the base editor was quickly implemented in generating *in vivo* models. For example, Katti et al. (2022b) developed a doxycycline-inducible base editing system to generate rapid *in vivo* models carrying disease-specific mutations in various organs, such as the lung, intestine, and pancreas. Moreover, base editing could achieve high mutational frequency in animal models compared to CRISPR nuclease (Rees and Liu, 2018), underscoring the potential of this tool in elucidating the function of disease-causing mutants in driving pathogenesis.

Despite its high efficiency, base editing cannot induce all SNVs and is currently restricted to six nucleotide changes. Moreover, it operates within a small window of 4–5 nucleotides, which may restrict the targetable bases, and, at the same time, results in unintended base conversions known as “bystander” mutations (Rees and Liu, 2018). However, an alternate approach known as prime editing was developed to overcome this limitation. Prime editing is a more versatile tool that enables all nucleotide changes as well as small indels (Anzalone et al., 2019). It achieves this by utilizing a unique molecular mechanism that combines Cas9 nickase and reverse transcriptase guided by prime-editing guide RNA (pegRNA). Within a short time, several studies have already demonstrated the ability of a prime editor to install desired mutations in cell lines, organoids, and mouse models (Chen and Liu, 2023; Ely et al., 2023). Li et al. (2022b) utilized a prime editor to generate isogenic induced pluripotent disease models. Another recent study showed prime editor’s efficiency in correcting mutations caused by large chromosome rearrangements. In this study, the authors employed a prime editor to replace a ∼1.38-kb sequence with a ∼19-bp sequence in *fumarylacetoacetate hydrolase* (*FAH*) gene in the liver. This replacement successfully restored FAH gene expression and hepatocyte repopulation in the liver, highlighting the potential of a prime editor in achieving complex gene editing (Jiang et al., 2022a). However, the efficiency of the machinery is low and prone to errors. Additionally, it can induce off-target effects that need to be further optimized. Nevertheless, these recent innovations offer a promising and safer alternative to traditional CRISPR techniques and may have significant implications in both basic cancer research and clinical applications.

## CRISPR as a potential therapeutic modality for cancer

Programmable gene-editing tools have made significant advancements in treating diseases [reviewed in detail in Katti et al. (2022a); Lattanzi and Maddalo (2022); Awwad et al. (2023); Li et al. (2023a)]. Adoptive T-cell therapy is one area of cancer research where CRISPR systems have shown immense therapeutic potential. They have been exploited to enhance the efficacy and potency of immunotherapy, especially in CART cell therapy. It involves *ex vivo* manipulation of patient T cells before infusing the cells back to the patient (Khalil et al., 2016a). For example, CRISPR has been employed to insert CAR in the T-cell receptor alpha constant (*TRAC*) locus and to delete several genes, including *PD-1*, to improve antitumor effect in various cancer models (Choi et al., 2019; Stadtmauer et al., 2020a). Several clinical trials utilizing this application are being tested (Stadtmauer et al., 2020a). While a pilot trial has demonstrated promising results, concerns over off-target edits and chromosomal rearrangements remain (Stadtmauer et al., 2020a). To overcome these issues, base editing is currently being evaluated as a potential therapeutic tool (Rees et al., 2021a). For instance, to reduce chromosomal translocations and promote efficient CART cell engineering, a combination of different CRISPR orthologs (SpCas9 and SaBE) is proposed to simultaneously knock-in and inactivate multiple genes (Glaser et al., 2023a). In addition to immunotherapy, attempts to directly modify cancer cells using CRISPR by targeting cancer-specific gene fusions or by directly correcting cancer driver mutations have been explored (Chen et al., 2017; Kim et al., 2018a). However, this is quite challenging considering the ever-evolving cancer mutation landscape. These preclinical studies demonstrate encouraging results, but extensive research is required to establish CRISPR as a feasible clinical therapy for cancer.

## CRISPR in cancer diagnostics

Due to its high precision and specificity, CRISPR is increasingly recognized as a potent nucleic acid-based detection tool in cancer diagnostics (Kaminski et al., 2021a). The endonuclease activity of the Cas protein has been leveraged to detect cancer-associated mutations in the patient sample (Lee et al., 2017; Nachmanson et al., 2018; Kaminski et al., 2021a). For instance, Lee et al. (2017) developed a method called CRISPR-mediated, ultrasensitive detection of target DNA (CUT)-PCR, which can detect minute amounts of circulating tumor DNA (ctDNA), a potential cancer-specific biomarker from patient blood, with remarkable sensitivity (<0.01%) and accuracy. This method selectively degrades the wild-type DNA sequence using CRISPR endonucleases (Cas9 and Cas12) and, subsequently, amplifies the mutant DNA by PCR followed by sequencing. Notably, this method has been successfully applied to detect oncogenic mutations in the ctDNA from the blood of colorectal cancer (CRC) patients (Lee et al., 2017). In a different study, the CRISPR/Cas system was employed to detect microsatellites, a cancer biomarker, by targeted degradation of short tandem repeats (STRs) followed by sequencing (Shin et al., 2017). Additionally, another study utilized a similar CRISPR/cas9-based fragmentation approach coupled with duplex sequencing (DS) that incorporates double-stranded molecular barcoding termed CRISPR-DS. This allows for an efficient enrichment of the target genomic region even from very low input DNA. This method is currently being tested in the clinic for the detection of oncogenic TP53 from the peritoneal fluid of women with ovarian tumors (Nachmanson et al., 2018).

Furthermore, several other techniques, such as specific high-sensitivity enzymatic reporter unlocking (SHERLOCK) and DNA endonuclease-targeted CRISPR trans reporter (DETECTR), utilize Cas12 or Cas13 proteins combined with isothermal amplification to detect nucleic acid from a single molecule of DNA or RNA. These systems offer the potential to detect patient-specific mutations in tumor biopsies with single-base mismatch specificity and high sensitivity (as low as 2 attomolar) (Chen et al., 2018; Gootenberg et al., 2018). For instance, Gootenberg et al. (2018) developed and applied the SHERLOCK platform to detect mutations in liquid biopsies from lung cancer patients. They reported a superior sensitivity of the system that can detect mutations as low as 0.1% of total DNA (Gootenberg et al., 2018). A recent study reported a Cas12-based one pot isothermal assay that combines endonuclease activity of Cas12a and rolling circle amplification for the sensitive detection of cancer miRNAs. This system was successfully implemented to detect miRNAs in pancreatic cancer patients at a femtomolar concentration with single-nucleotide specificity (Yan et al., 2023). These advancements highlight the potential of the CRISPR/Cas system as a cancer-specific biomarker detection tool. With ongoing developments in the field, CRISPR could soon become a highly sensitive and personalized diagnostic tool for patients with cancer.

## CRISPR/Cas9 in other diseases

Apart from cancer, CRISPR/Cas9 has had a profound impact on other diseases (Li et al., 2023). For instance, CRISPR holds a great promise in treating hemoglobinopathies such as sickle cell disease (SCD) and beta thalassemia. These are genetic disorders caused by mutations in the beta globin (*HBB*) gene (Frangoul et al., 2020). CRISPR knockout and CRISPRi screening identified stage-specific, lineage-restricted enhancers for the *BCL11A* gene, which is a transcription repressor of fetal hemoglobin (HbF). The depletion of BCL11A derepresses HbF gene expression and alleviates beta-hemoglobinopathies (Bauer et al., 2013; Canver et al., 2015). At present, CRISPR-based gene knockout and base editor-mediated silencing of the BCL11A enhancer are actively being tested as a precision genome-editing therapy in the clinic (Khosravi et al., 2019; Frangoul et al., 2020).

CRISPR-mediated gene editing can be used to treat cardiovascular diseases such as hypercholesterolemia. For instance, Musunuru et al. (2021) and Rothgangl et al. (2021) used base editing to introduce precise loss-of-function mutation in PCSK9 using lipid nanoparticle. They showed that single administration of base editors resulted in a long-term reduction of the PCSK9 protein and low LDL levels in monkeys, showing a promising strategy in treating high blood cholesterol levels in humans. Interestingly, when this strategy was tested in clinics, it showed a superior effect compared to currently approved drugs, thus underscoring the importance of CRISPR gene editing in treating cardiovascular diseases (Musunuru et al., 2021; Rothgangl et al., 2021).

In addition, CRISPR/Cas9 technology is widely used to treat diseases, such as Alzheimer’s disease and obesity, and other genetic disorders, such as muscular dystrophy and cystic fibrosis (Matharu et al., 2019; Maule et al., 2021; Bhardwaj et al., 2022; Li et al., 2023).

## Limitations

Although CRISPR technology has immense potential in accelerating drug discovery and development, several challenges need to be addressed. For instance, off-targets and inefficient editing could compromise the specificity and potency of CRISPR-mediated gene editing. These limitations can be mitigated by improving the sgRNA design and engineering Cas9 variants with high specificity and minimal off-target effects (Slaymaker and Gaudelli, 2021). Moreover, although CRISPR technology is a valuable tool for rapidly generating somatically engineered mouse models (SEMMs) (Lima and Maddalo, 2021), it is quite challenging to efficiently deliver CRISPR components and precisely edit specific cell and tissue types *in vivo*. This can be overcome by the delivery of CRISPR components using viral vectors. In an ideal scenario, an efficient delivery system should be specific to the target site and elicit low immunogenicity. While delivery via AAV vectors proves to be effective and offers several benefits, including its non-integrative nature, low immunogenicity, and broad tissue tropism, they are expensive and offer limited payload capacity (∼4.5 Kb), making the delivery of the CRISPR machinery and sgRNA challenging (Wang et al., 2019). For instance, it is not possible to use AAV to deliver epigenome, base, or prime editors. One way to overcome this challenge is to deliver the CRISPR machinery using dual AAV split systems. Several recent studies have successfully utilized split systems to deliver CRISPR machinery in various organs in mouse models (Levy et al., 2020; Davis et al., 2023). An alternative approach is to utilize Cas9 from other species, such as SaCas9, which are smaller and favor *in vivo* gene transfer (Hendriks et al., 2020). Currently, non-viral delivery of nucleic acids using lipid nanoparticle offers a great alternative and is currently being tested in clinical trials following its success in delivering mRNA-based COVID-19 vaccines (Thi et al., 2021). While these studies are promising, further improvements in both delivery approaches and minimizing off-target effects are needed to make CRISPR a viable tool in cancer therapy.

## Conclusion and future perspectives

Within a decade of its implementation in mammalian cells as a gene editing tool, CRISPR technology has substantially expanded the scope of genetic research, enabling us to manipulate DNA with great precision and ease. This groundbreaking technology has not only enabled us to address genetic diseases but also opened up new avenues through epigenome editing for tackling non-genetic diseases. Additionally, recent advancements in base and prime editors have greatly improved our ability in modeling and characterizing disease-associated mutations and will be instrumental in identifying novel therapeutic targets and understanding resistance mechanisms. These efforts will undoubtedly facilitate the development of precision medicine-based approaches to treat different cancer types. CRISPR holds immense promise for the future, as ongoing advancements in the field will continue to propel novel findings that accelerate drug discovery, treatment, and diagnosis.
